# Copper-Silver Nanohybrids: SARS-CoV-2 Inhibitory Surfaces

**DOI:** 10.3390/nano11071820

**Published:** 2021-07-13

**Authors:** Dina A. Mosselhy, Lauri Kareinen, Ilkka Kivistö, Kirsi Aaltonen, Jenni Virtanen, Yanling Ge, Tarja Sironen

**Affiliations:** 1 Department of Virology, Faculty of Medicine, University of Helsinki, P.O. Box 21, 00014 Helsinki, Finland; lauri.kareinen@helsinki.fi (L.K.); ilkka.kivisto@helsinki.fi (I.K.); kirsi.aaltonen@helsinki.fi (K.A.); jenni.me.virtanen@helsinki.fi (J.V.); 2 Department of Veterinary Biosciences, Faculty of Veterinary Medicine, University of Helsinki, P.O. Box 66, 00014 Helsinki, Finland; 3 VTT Technical Research Center of Finland Ltd., P.O. Box 1000, 02044 Espoo, Finland; Yanling.Ge@vtt.fi

**Keywords:** SARS-CoV-2, human-animal interfaces, nanohybrids, copper, silver, inhibitory surfaces

## Abstract

The severe acute respiratory syndrome coronavirus 2 (SARS-CoV-2) remains a severe health threat. The COVID-19 infections occurring in humans and animals render human-animal interfaces hot spots for spreading the pandemic. Lessons from the past point towards the antiviral properties of copper formulations; however, data showing the “contact-time limit” surface inhibitory efficacy of copper formulations to contain SARS-CoV-2 are limited. Here, we show the rapid inhibition of SARS-CoV-2 after only 1 and 5 min on two different surfaces containing copper-silver (Cu-Ag) nanohybrids. We characterized the nanohybrids’ powder and surfaces using a series of sophisticated microscopy tools, including transmission and scanning electron microscopes (TEM and SEM) and energy-dispersive X-ray spectroscopy (EDX). We used culturing methods to demonstrate that Cu-Ag nanohybrids with high amounts of Cu (~65 and 78 wt%) and lower amounts of Ag (~7 and 9 wt%) inhibited SARS-CoV-2 efficiently. Collectively, the present work reveals the rapid SARS-CoV-2 surface inhibition and the promising application of such surfaces to break the SARS-CoV-2 transmission chain. For example, such applications could be invaluable within a hospital or live-stock settings, or any public place with surfaces that people frequently touch (i.e., public transportation, shopping malls, elevators, and door handles) after the precise control of different parameters and toxicity evaluations.

## 1. Introduction

There are public and scientific demands to unravel where we currently stand in our fight against COVID-19. Is the world prepared to combat another wave of the severe acute respiratory syndrome coronavirus 2 (SARS-CoV-2)? From a surface disinfection point of view, how long can SARS-CoV-2 survive on surfaces, and how fast can SARS-CoV-2 particles be disinfected from these surfaces to cut the chain of infection? Could nanoparticles (NPs) constitute promising broad-spectrum antiviral (anti-SARS-CoV-2) arsenals?

Let us answer the questions mentioned above in a narrative, starting from the current situation of the COVID-19 pandemic. On 31 January 2021, the World Health Organization (WHO) updated the total number of COVID-19 cases to over 102 million, and the number of deaths to 2.2 million worldwide. The WHO also mentioned that Saturday, 30 January 2021, marked one-year since its declaration of COVID-19 being a Public Health Emergency of International Concern [1]. When it comes to COVID-19 animal cases within 2021, several species have been reported to be affected, including puma (in Argentina), lions (in Estonia), dogs (in Bosnia and Herzegovina), minks (in Poland), and cats (in Latvia) [2]. Earlier in the spring of 2020, mink farms were drastically hit with SARS-CoV-2 outbreaks in several countries, and both human-to-mink and mink-to-human transmission [3]. What lay at the end of 2020 was the massive culling of over 2.7 million minks in the Netherlands after a farm outbreak, amounting to more than 6.5 times the number of confirmed human cases [4].

On the vaccination side, the BioNtech/Pfizer and Moderna vaccines with mRNA-based NP formulations are the forerunner vaccines. They use lipid NPs (spherical ionizable lipid vesicles that are positively charged at a low pH, facilitating RNA complexation, and neutral at a physiological pH, reducing side toxic effects [5]) as carriers, allowing endosomal escape. This formulation allows the stable and efficient release of the genetic cargo to the host cell cytosol, initiating the synthesis of SARS-CoV-2 spike proteins that induce the production of neutralizing antibodies by the immune system [6]. We previously recommended parallel vaccination strategies for humans and animals, mainly minks, raccoon dogs, cats, and zoo animals, using the NP BNT162b2 (BioNtech/Pfizer) and mRNA-1273 (Moderna) vaccine formulations [7]. Recent work by Hoffmann et al. [8] investigated antibody-mediated neutralization based on the S protein using vesicular stomatitis virus (VSV)-based vectors pseudotyped with several variant-specific SARS-CoV-2 S proteins. The group used the following SARS-CoV-2 variants: (i) UK variant (B.1.1.7, namely the variant of concern (VOC) 202012/01 or 20I/501Y.V1); (ii) South African variant (B.1.351, namely 20H/501Y.V2); and (iii) Brazilian variant (B.1.1.248, namely P.1.). The work indicated that some of these variants might evade the antibody response, showing the reduced neutralization of the South African and Brazilian variants compared to the neutralization of SARS-CoV-2 WT (Wuhan-1 isolate), raising concerns that convalescent SARS-CoV-2 WT patients may be only partially protected against the South African and Brazilian SARS-CoV-2 variants. The work rang another alarm bell by showing inadequate protection against these variants in sera from donors who had received two doses of the BNT162b2 (BioNtech/Pfizer) vaccine. This lack of protection occurs because, while the vaccination completely inhibited viral entry by SARS-CoV-2 WT, the inhibition was reduced for the UK variant and almost entirely absent for the South African and Brazilian variants [8]. Would the universal administration of NP-containing, self-disinfecting surfaces overcome the SARS-CoV-2 vaccination challenges?

There are still knowledge gaps about the transmission routes of SARS-CoV-2, but research indicates that airborne routes, direct contact, droplets, and fomites may all be involved [9]. However, the significance of transmission through these routes remains unclear, especially when it comes to surface-mediated transmission. SARS-CoV-2 RNA has been detected from surfaces around patients in hospital rooms, but the challenges in culturing the virus from the environment make it difficult to determine whether such findings are clinically relevant [10,11]. In studies performed within laboratory settings, the surface stability of SARS-CoV-2 was variable depending on the surface type and many environmental factors. SARS-CoV-2 may remain infectious on surfaces for durations ranging from several days (e.g., on plastic, steel, and fur) to as little as a few hours (e.g., on wood, paper, and cloth), and it is inactivated more rapidly at higher temperatures and humidity [12,13]. For example, SARS-CoV-2 has demonstrated extreme stability in a wide range of pH values (pH 3 to 10) at room temperature [14]. When comparing stability on hard surfaces, SARS-CoV-2 is more stable on plastic and stainless steel (viable virus detected for up to 72 h) than on cardboard and copper (viable virus detected for up to 24 h and 4 h, respectively) [15]. SARS-CoV-2 nano-inhibitory surfaces could then be a tool (e.g., wearing masks that could reduce the amount of infectious virus, ventilation, hand hygiene, reducing crowding and indoor gatherings, [9,11] and vaccination strategies) within the game-changer toolbox, containing the indirect transmission of COVID-19 via surfaces without pharmaceutical intervention.

Previous research has established the efficient role of copper ions Cu(I) and Cu(II) on alloys with 60% Cu in the inactivation of murine norovirus-1 (MNV-1, a surrogate for human noroviruses) [16]. Data from other research recognize the antiviral properties of hybrid coatings containing silver (Ag), Cu, and zinc (Zn) cations against human immunodeficiency virus type-1 (HIV-1) with 99.5% titer reduction after 20 min of exposure, and the potential application of the coatings for viral inhibition on surfaces [17]. An observational pilot study investigated copper’s antiviral efficacy against healthcare-related infections in nursing homes (influenza A and norovirus, which cause influenza and gastroenteritis outbreaks, respectively). The study compared facility wards equipped with copper surfaces (i.e., 90% copper on door handles, handrails, and grab-bars) vs. those not equipped with them. The study revealed copper’s ability to reduce hand-transmitted healthcare-associated infections [18].

Turning now to the fascinating anti-SARS-CoV-2 NP solutions, recent research has shown the potential of nano-based antiviral agents to inhibit SARS-CoV-2, specifically in the air, on surfaces, and in personal protective equipment. However, such SARS-CoV-2 NP disinfectants’ practical use remains in the early stages due to the lengthy and strict regulatory and toxicological assessments required for such applications [19]. The promising antiviral capabilities of NPs stem from their ability to generate reactive oxygen species (ROS) and their photodynamic and photothermal properties [19]. A group of researchers showed in a preprint [20] that Luminore CopperTouch^TM^ surfaces inactivated 99% of SARS-CoV-2 particles after 2 h, and recommended administering such surfaces within hospital and public transportation settings to reduce viral spread. Interestingly, Balagna and colleagues [21] demonstrated the inhibition of SARS-CoV-2 (the absence of cytopathic effects on cell culture and a complete SARS-CoV-2 titer reduction to zero) on a sputter-coated FFP3 mask (3M™) by Ag nanocluster-silica composite coating (<200 nm with Ag 1.53 at%).

**Figure 1:**
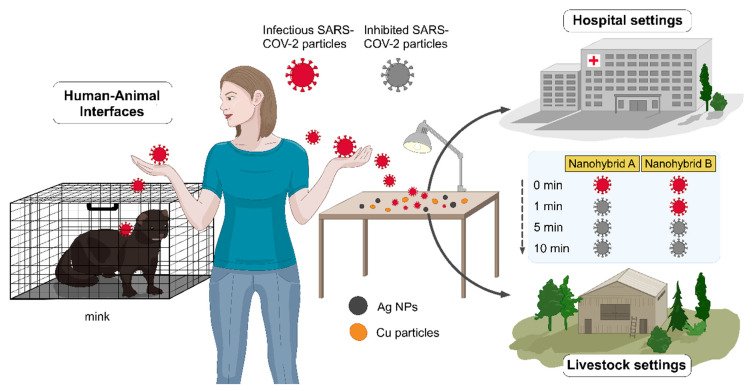
Out-of-the-box surface administration of Cu-Ag nanohybrids rapidly inhibits SARS-CoV-2 (after 1 and 5 min), breaking the SARS-CoV-2 transmission chains and containing the pandemic within the hospital and livestock settings, and in public reservoirs. Nanohybrids A and B represent samples 2 and 3, containing ~65 and 78 wt% Cu and ~7 and 9 wt% Ag, respectively.

The complex mechanisms of SARS-CoV-2 transmission via droplets and small aerosols landing on surfaces and remaining infectious for varying times make an assessment of environmental transmission chains challenging. Here, we studied novel ways of inactivating SARS-CoV-2 on surfaces that could be deployed in public places, people’s homes, and health care settings (as seen in Figure 1). We investigated the usefulness of Cu-Ag nanohybrid powders and plated surfaces in inactivating SARS-CoV-2 to evaluate the potential of such products to contain the pandemic. We also examined the characteristic parameters affecting the SARS-CoV-2 inactivation for future surface applications.

## 2. Results and Discussion

### 2.1. Characterization of Copper-Silver Nanohybrids’ Samples

Clean Touch Medical LTD is developing antimicrobial nanohybrid surfaces and delivered several samples to the University of Helsinki (details given in the Methods section below) for testing their inhibitory effects against SARS-CoV-2. We also collected the raw powder material from the Lainisalo Industrial Painting factory. We thoroughly characterized the powder material using microscopy tools to ensure the properties of the hybrid samples. Afterward, we tested the provided samples for surface SARS-CoV-2 inhibition. Only two surfaces showed viral inhibitions, and even the surface primarily formed from the collected powder sample did not inhibit SARS-CoV-2. Consequently, we characterized parallel samples of the tested surfaces by SEM to draw a concrete conclusion on the reasons governing the inhibitory effects. The Cu-Ag nanohybrid powder collected (sample P) and the plated surfaces (samples 2 and 3) were characterized by a series of microscopy techniques, namely transmission electron microscope (TEM) for imaging and electron diffraction ring pattern, high-resolution TEM (HRTEM), scanning electron microscope (SEM), and energy-dispersive X-ray spectroscopy (EDX) in the scanning transmission electron microscope (STEM) and SEM.

**Figure 2:**
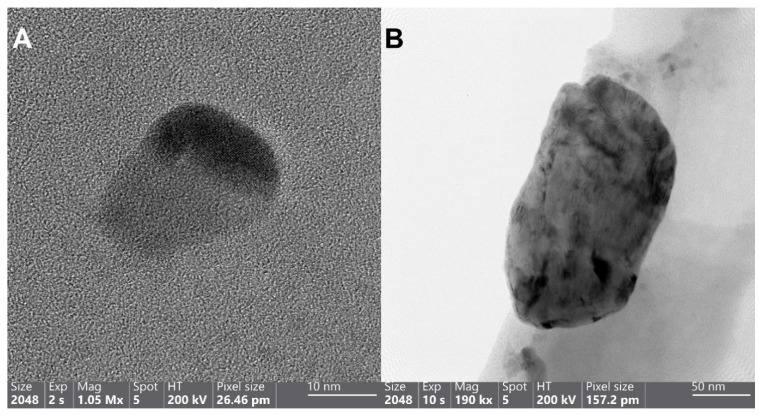

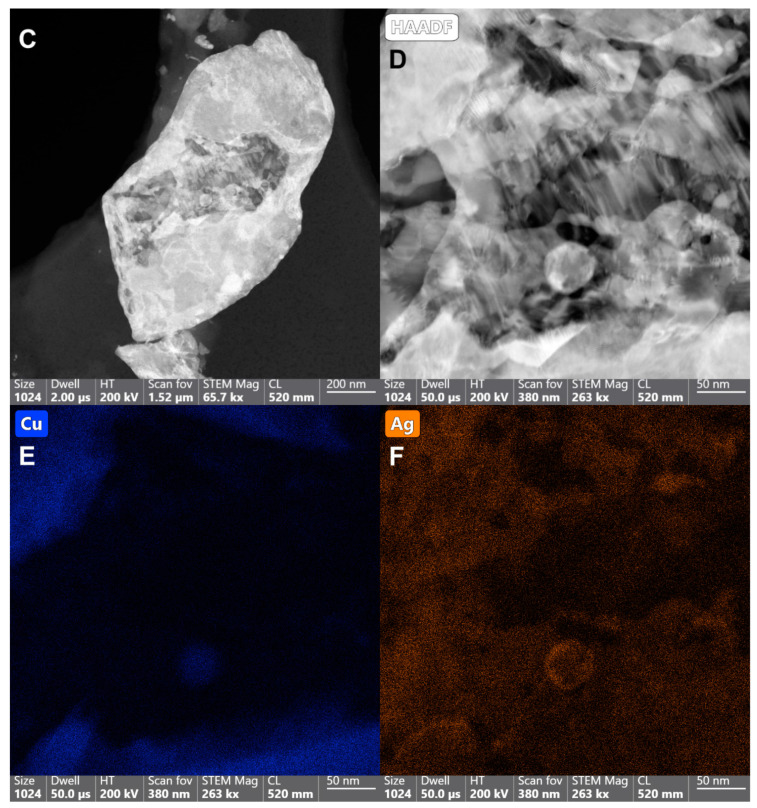
HRTEM (**A**) and TEM (**B**) images of the Cu-Ag nanohybrid sample P, depicting the wide differences in the size of Ag NPs spanning from as small as 26 nm (**A**) to as large as 212 nm (**B**). HAADF images ((**C**,**D**) “as a close-up of panel C”) and the corresponding maps of panel D, demonstrating the allocated Cu and Ag places with blue (**E**) and orange colors (**F**), respectively.

TEM imaging, analysis, and mapping (Figure 2 and Figure S3) show the different particle sizes, shapes (irregular rounded, rectangular, rectangular embracing smaller spherical particles, and flakes), distribution, composition, and the crystalline structure of particles within sample P. Regarding the particles’ size, the diameter of each particle has been measured five times from different angles, as they are not perfectly shaped. Figure 2A,B depicts the size of ~26 ± 2 nm irregular rounded Ag NPs and ~212 ± 16 nm irregular rectangular Cu NPs, respectively. Figure 2C,D demonstrates the large-sized (i.e., in the micrometer range) irregular sharp-edged rectangular copper particles ~1.3 ± 0.2 µm containing the smaller ~51 ± 2 nm spherical Ag NPs with their mapped blue (panel E) and orange colors (panel F), respectively. The selected-area electron diffraction (SAED) ring pattern of sample P is a mixture of two face-center cubic patterns. The *d* values calculated correspond to the plane spacing of Ag and Cu NPs’ crystal structures reported in the International Center for Diffraction Data (ICDD 01-087-0720 of Ag and ICDD 01-085-1326 of Cu) and are depicted in Figure S3 (panel B).

**Figure 3:**
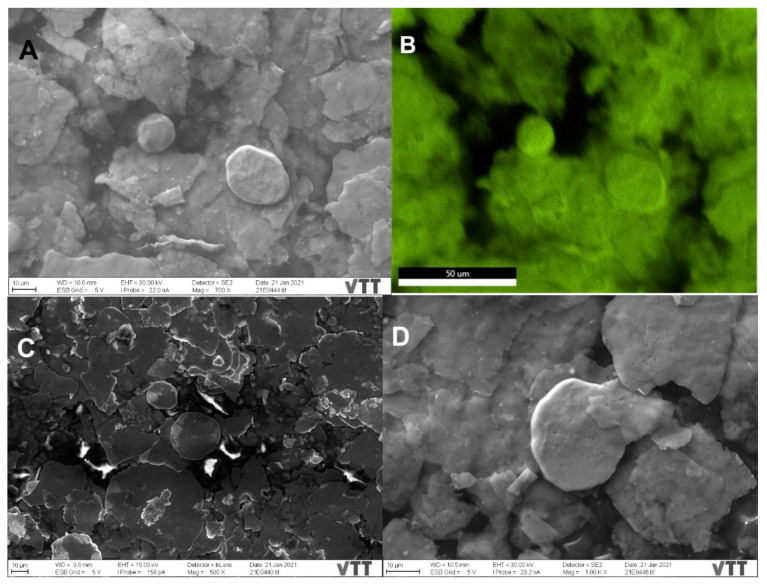

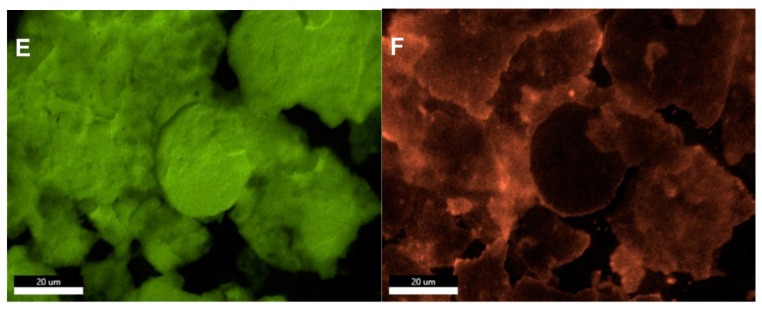
SEM images depicting the surface morphology of samples 2 and 3 with irregular flakes and rounded, moon-shaped particles, with minor rounded details on top. (**A**) Sample 2 with the mapped Cu NPs demonstrating their fluorescent green color (**B**). Images of sample 3 using the in-lens detector, with charging effects, (**C**) and secondary electron detector, with reduced charging effects (**D**). The Cu (**E**) and Ag (**F**) distribution in the selected map are displayed in their fluorescent green and orange colors, respectively, implying that the moon-shaped particles are also Cu.

SEM imaging and analysis reveal the surface morphology and the structure of the plated surfaces, namely sample 2 and sample 3. Unfortunately, sample 1 suffered charging effects and even burning under the SEM, making a hole in the sample even after decreasing the accelerating voltage from 30 kV to 5 and 2 kV, rendering it difficult for imaging and analysis. Based on information provided by the manufacturing company, sample 1 is a mixture of sample P (which has been thoroughly characterized above by TEM) and paint, (which could be organic based on the reported charging effects), combined and then plated on the surface. Sample 2 (Figure 3A) shows the different morphologies spanning from flakes to rounded particles, taking a moon shape (16 ± 0.6 µm size) or irregular spherical shape (28 ± 1 µm size), with minor rounded details. The surface of sample 2 is also not flat, displaying different levels or layers, and roughness. The mapping of panel 3A demonstrates that the moon and irregular spherical particles are Cu NPs, displaying a fluorescent green color (Figure 3, panel B) and not Ag NPs (Figure S4). Sample 3 (Figure 3C,F) displays mostly the same surface morphology (flakes and rounded moon-shape particles with the top featuring minor rounded details) as sample 2 with some differences, including: (i) The observed greater charging effects in sample 3 (Figure 3C) while using the in-lens detector. Therefore, the detector has been changed to the secondary electron to eliminate these charging effects (Figure 3D); (ii) The size of Cu NPs (19 ± 0.8 µm the moon-shaped and 31 ± 0.8 µm size, Figure 3C) is slightly larger than that of sample 2; (iii) The surface of sample 3 is smoother than that of sample 2 (i.e., topologically, the surface of sample 3 can be characterized as less rough). Such a smooth surface could theoretically hinder the rapid surface contact interaction between Cu NPs and SARS-CoV-2 particles.

Interestingly, the quantitative chemical composition of surface samples 2 and 3 is indicated by the EDX. Sample 2 EDX quantitative results infer that the highest concentration was reserved for the Cu (~65 wt%), and the Ag constitutes a low concentration (~7 wt%) as outlined in Table S1. Other elements were also detected in variable quantities (Si, Al, Ti, Sn, Cl, and Na), with the lowest element detected having been Si (~1 wt%), and the second-highest element detected was Na (~13 wt%). Sample 3 EDX results indicate that the highest concentration was of Cu (~78 wt%), as outlined in Table S2. The Ag comprises a concentration of only (~9 wt%) with both Cu and Ag sample 3 concentrations detected being higher than that of sample 2. Besides, other elements were also detected within the composition of sample 3 in minor amounts, including Fe, Ti, and Sn (which could be considered impurities), and Si and Al (which could be elicited from the SEM detector and the electron column, respectively). Moreover, the reader should keep in mind that Tables S1 and S2 do not include the light elements since EDX is not a reliable tool for quantifying light elements.

### 2.2. Anti-SARS-CoV-2 Effects at Surface Exposure

SARS-CoV-2 is an enveloped, positive-sense single-stranded RNA virus, sharing a number of characteristics with other RNA respiratory viruses (SARS-CoV-1 and MERS-CoV coronaviruses, and H1N1, H5N1, and H5N7 influenza viruses). These shared characteristics include their zoonotic origins and later adaptations for better human-to-human transmission by direct (via mouth, nose, and eyes) or indirect contact (via contaminated surfaces or equipment) [22].

**Figure 4:**
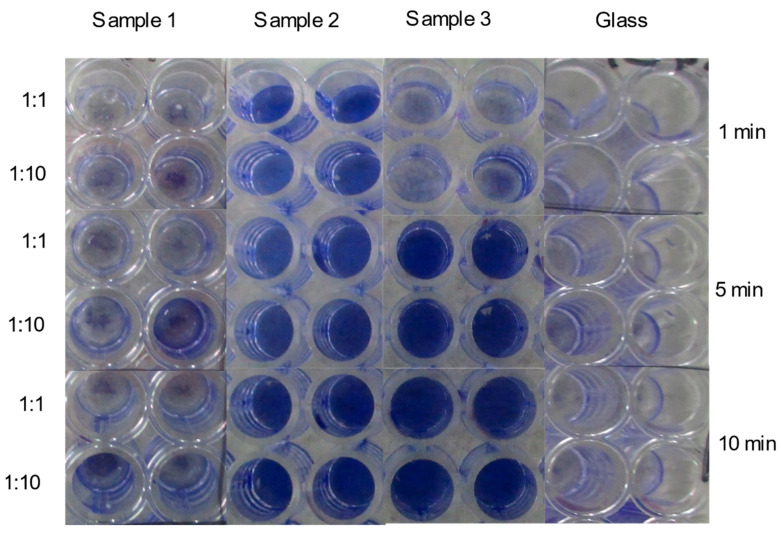
A composite image showing inhibitory effects of Cu-Ag nanohybrid powder sample (sample 1) and plated surfaces (samples 2 and 3) on the growth of SARS-CoV-2 after time points 1 min, 5 min, and 10 min, based on crystal violet-stained Vero E6 cells. The blank color indicates virus growth and the violet color indicates an inhibitory effect.

The EDX quantitative results (Tables S1 and S2) delineate that the principal chemical element of the plated surfaces used in this study was Cu and its particulate form. Therefore, any antiviral effects are primarily attributed to copper, especially in samples 2 and 3. In this study, the surface inhibitory effects on SARS-CoV-2 were assessed by inoculating virus stock onto surfaces coated with Cu-Ag nanohybrids using glass as a non-reactive control surface (Figure 4, Table S3). These surfaces were sampled at three different time points (1, 5, and 10 min) by rubbing with a moistened cotton swab with cell culture media. The swab samples were briefly vortexed in the media, and the supernatant inoculated on Vero E6 cells at two dilutions (1:1 and 1:10). After inoculation, the cells were incubated for four days in +37C, fixed, stained, and assessed for cytopathic effect (CPE). With samples 1 and glass, CPEs were detected at all sample time points and in both culture dilutions, while in sample 3, CPEs were observed at 1 min but not at 5 or 10 min. No CPE was observed in sample 2 at any of the time points tested.

There have been some previous studies on the effect of Cu on RNA respiratory viruses. Fujimori et al. [23] demonstrated the antiviral effects of copper(I) iodide NPs of 160 nm size against H1N1 influenza A virus, using plaque titration assay with a 1 h contact time. They attributed the antiviral effects to the role played by both the produced hydroxyl radicals, inactivating the viral hemagglutinin (HA) and neuraminidase proteins, and the Cu^+^ oxidizing the lipids of the enveloped H1N1, inactivating it. Minoshima et al. [24] indicated the superior inhibitory effect against H1N1 of solid-state cuprous oxide (Cu_2_O) compared with Ag_2_S particles after 30 min. In the same vein, they assigned the H1N1 inhibitory effects to the denaturation of the viral HA protein interfering with host cell viral recognition. More recently, Das Jana and colleagues [25] showed the inhibitory effects of copper oxide (Cu_2_O) NPs in a composite with graphene sheets on influenza A virus after 30 min of contact. They ascribed the antiviral effects of Cu_2_O NPs to their interference with the structure and the function of viral HA, disrupting the viral structural integrity to enter host cells, and consequently preventing viral replication and infection.

Across the SARS-CoV-2 surface stability literature, vast differences were detected in the SARS-CoV-2 inactivation associated with different surfaces, environmental conditions, and laboratory settings. Therefore, there is a pressing need for consensus on standardized surface testing protocols in order to safeguard accurate SARS-CoV-2 surface data comparability, so that an efficient intervention to break the transmission chains can be achieved [12].

To ensure that the CPE was elicited by SARS-CoV-2 and not by any cytotoxic substances dissolved from the test surfaces, culture media were tested with RT-PCR (Table 1 and Table S4). Samples were reported positive for viral growth if at least one of the parallel reactions with either dilution had visible CPE and the Ct (cycle threshold)-value was below 20. Ct-values above 30 were considered to be caused by the original inoculum and not viral growth (Figure S5). RT-PCR results confirmed that sample 3 inhibited the viral growth from 5 min onwards, and sample 2 already inhibited the growth at the one-minute time point. No inhibitory effects were detected with sample 1 or the negative control. It is essential to bear in mind that these results describe viral inhibition, preventing the SARS-CoV-2 from infecting the cells. Furthermore, the most remarkable aspect of the results is the rapid onset of inhibitory effects in samples 2 and 3. The inhibitory effect is detectable even when using very high viral concentrations unlikely to occur in natural infection settings.

The efficient SARS-CoV-2 inhibition by the present Cu-Ag nanohybrids in less than 5 min is noteworthy because such rapid inhibition has not previously been described. Cu formulations in other studies have shown the inhibition of influenza A after 30 min [24,25], 1 h [23], or even 6 h [26]. On 29 February 2008, the EPA registered five copper-containing alloy products, allowing them to state in their marketing that they “killed 99.9% of pathogenic bacteria after 2 h” [27].

Hutasoit and colleagues [28] detected a 96% inactivation of SARS-CoV-2 on stainless steel coated with Cu in 2 h. Other recent reports have shown Cu formulations inactivating SARS-CoV-2 in less time, with the shortest inactivation times being 1 h (using Cu_2_O particles bound with polyurethane [29]) to 30 min (by a spray-coated aqueous colloidal dispersion of poly(ionic liquid)/Cu composite [30]). An implication of Cu-Ag nanohybrids’ fast surface inhibitory effect is that the administration of these substances on key surfaces could potentially provide a practical, effective barrier to virus transmission.

Considering the efficient SARS-CoV-2 inhibition by the present Cu-Ag nanohybrids in less than 5 min, it remains to be seen whether such compounds could be employed to reduce the burden of disease caused by these devastating infections.

In the present study, the chemical composition of samples 2 and 3 indicates that some impurities (e.g., Na, Cl, Sn, Fe, and Ti) remain and should be excluded after the optimization of the synthesis procedure to minimize possible toxicity. Also, the size distribution detected for the Cu NPs from sample P is considerable, ranging from the nanometer range to the micrometer range after platting onto surfaces (samples 2 and 3). Most of the previously reported anti-CoV NPs were within the nanometer range [31], including the Ag_2_S nanoclusters (only 3 and 4 nm) that inhibited the porcine epidemic diarrhea virus via inhibiting the viral negative-strand RNA synthesis and viral replication [32]. There are many unanswered questions about the in vitro toxicity of such materials when in contact with skin cells. In this regard, natural anti-SARS-CoV-2 products could limit the concerns about in vivo usage, which remain to be thoroughly studied in all such surface inhibitory applications. Selwyn and colleagues [33] showed SARS-CoV-2 inactivation after 5, 20, and 60 min using a combination of a natural citric acid with 3% thymol and 1297 quaternary ammonium compound which was infused in surgical masks.

A key challenge in the toxicity assessment of SARS-CoV-2 inhibitory NPs are the multiple chemical and physical parameters affecting the composition and the structure of these particles [7]. Therefore, a precise manufacturing process to minimize the size variations of Cu and Ag NPs that were seen in this study should be employed to investigate the anti-SARS-CoV-2 activity further. Elucidating the precise mechanisms behind the anti-SARS-CoV-2 inhibitory effects of Cu NPs, Ag NPs, and Cu-Ag nanohybrids, could provide a better understanding of the SARS-CoV-2- metal interactions, and thorough toxicity assessments are warranted. However, the nanohybrid materials already provide a powerful candidate for effective and easy physical infection barriers.

## 3. Conclusions and Outlook

In the current work, we demonstrate the efficient, rapid inhibition of SARS-CoV-2 after only 1 and 5 min on two different surfaces containing copper-silver (Cu-Ag) nanohybrids. We attribute the role of primary SARS-CoV-2 inhibition to Cu, which constituted the highest proportions in the two inhibitory surfaces tested. We propose coating such surfaces with Cu-Ag nanohybrids to break the SARS-CoV-2 transmission chains within hospital and live-stock settings and in public places, after thorough toxicological evaluation and optimizing the synthesis procedure and parameters of the nanohybrids.

Our future work is directed towards unveiling the molecular mechanisms governing the inactivation of SARS-CoV-2 by such Cu-Ag nanohybrid surfaces, and their role in fighting the new SARS-CoV-2 variants and other pathogens with the ability to spread through contaminated surfaces, such as methicillin-resistant *Staphylococcus aureus* and human noroviruses.

## 4. Methods

### 4.1. Copper-Silver Nanohybrid Powders and Plated Surfaces

The Cu-Ag nanohybrid powder sample (sample P) was collected from the Lainisalo Industrial Painting factory (wet-painting factory, Tallinn, Estonia) on 24 October 2020. The plated surfaces (samples 1, 2, and 3; Figure S1) were provided by the Clean Touch Medical LTD to the University of Helsinki to be tested to inhibit SARS-CoV-2 on 8 December 2020 with information that the samples were metal hybrid combinations of copper and silver. The surface coating of the samples was adopted using one of the two processes, namely powder coating (the coating was applied electrostatically and melted in the oven at 200 °C for 30 min) and wet painting (the coating was plated on the surface using a spray). The surface coating was accomplished on stainless-steel substrates, forming an even thickness of 40 µm.

### 4.2. Characterization of Copper-Silver Nanohybrids’ Samples

To assess how the powder sample and plated surfaces would inactivate the tested SARS-CoV-2, the samples’ structure and composition were thoroughly investigated via TEM and SEM.

For the powder sample (sample P) preparation, water was first used as a solvent. However, the sample was hydrophobic, and the powder painted the vial wall (Figure S2). Sample P was then dissolved in ethanol overnight. The shape and distribution of Cu and Ag particles in sample 1 were detected by a TEM (FEI TALOS F200X, Thermo Scientific™, Netherlands) operated at 200 kV accelerating voltage. The electron diffraction ring pattern confirmed the Cu and Ag particles’ crystal structure, and the morphology was investigated by the HRTEM. The chemical structure of sample 1 was detected by making two maps (each lasted 1 h) from the same position containing the ring pattern diffraction, using the EDX unit of the STEM.

For the plated surfaces samples preparation (samples 1, 2, and 3), the samples were placed on aluminum stubs and air blown. Then the samples were analyzed for their surface morphology and chemical composition with an SEM (Zeiss Crossbeam 540, Zeiss, Germany) operated using 15 and 30 kV acceleration voltage for imaging and EDX (each map lasted 1 h), respectively, using an in-lens detector. For sample 3, the in-lens and secondary electron detectors were used. The size distributions of the Cu and Ag particles on the obtained TEM and SEM images were analyzed using the ImageJ software (National Institutes of Health, Bethesda, MD, USA).

### 4.3. Viral Strain, Cell Culture, Anti-SARS-CoV-2 Surface Exposure Tests

Vero E6 cells were cultured in minimal essential Eagle’s medium (MEM, Sigma-Aldrich, Saint Louis, USA) supplemented with fetal bovine serum (FBS, Gibco; 10% for maintenance and 2% for infection), L-glutamine, penicillin, and streptomycin. The cells were infected with the SARS-CoV-2/Finland/1/2020 strain (passage 7) and incubated at 37 ± 2 °C with 5% CO_2_.

The aliquots of 25 µL SARS-CoV-2 viral suspension (50,000 PFU/mL) were spread on different Cu-Ag nanohybrid plated surfaces for 1, 5, and 10 min. Glass surfaces were used as a negative surface control. Afterward, surfaces were sampled with pre-wetted cotton swabs and placed in 500 µL MEM media, from which viral dilutions were prepared.

Vero E6 cells were infected with two serial dilutions, namely 1:1 and 1:10, in the 96 well plates for 1 h at +37 °C. The media were then changed to fresh MEM, and the cells were incubated for four days. Positive control viral dilutions and negative control MEM were included in the performed experiments. Two separate experiments were executed on different days, and each experiment was performed in duplicate.

All the SARS-CoV-2 experiments were performed in a BSL-3 laboratory at the Faculty of Veterinary Medicine, University of Helsinki. A video illustrating the execution of the anti-SARS-CoV-2 surface exposure tests in the BSL-3 facility is displayed in the supplementary material (Video S1).

#### 4.3.1. Cytopathic Effects (CPE)

After the 5-day incubation of the infected cells, the plates were investigated under an inverted microscope (CKX41, Olympus Life Science Corporation, Japan) to detect any cytopathic effects induced by the viable SARS-CoV-2 viruses. Samples were reported as positive for the infectious SARS-CoV-2 virus if CPE was observed. The visualization of the CPE was performed via the following series of steps. The cells of all the tested plates were: (i) Fixed (adding 100 µL of 37 wt% formaldehyde/well for 30 min); (ii) Washed with water (100 µL/well); (iii) Stained with crystal violet (50 µL of 1:5 diluted crystal violet solution for 10 min); (iv) Washed with water (100 µL/well). Violet stained cells indicated viral inhibition. Clear wells indicated the presence of the viable virus, infecting cells that washed away after the staining. The stained plates were then photographed by a digital camera.

#### 4.3.2. Real-Time Reverse-Transcription Polymerase Chain Reaction (RT-PCR)

Following the 4-day incubation, all cell culture media with the tested inactivation time points of 1, 5, and 10 min were transferred to new plates for RNA isolation and subsequent RT-qPCR to confirm the exclusive induction of reported CPE by the SARS-CoV-2 or the inactivation of SARS-CoV-2.

RNA was extracted from cell culture media with QIAcube HT (Qiagen, Germany) using the QIAamp 96 Virus QIAcube HT kit (Qiagen, Germany), following the kit protocol with off-board lysis. The protocol was as follows: 200 µL of the sample was added to 160 µL of ACL lysis buffer with carrier RNA and 20 µL of Proteinase K in BSL-3. After 30 min of incubation at room temperature, the surfaces of lysis blocks containing the lysed samples were wiped and sprayed with 80% ethanol. They were transferred outside of the BSL-3 for the rest of the isolation protocol. RT-qPCR targeting the SARS-CoV-2 N gene was used according to the procedure of Corman et al. [34]. Samples were considered positive for SARS-CoV-2 infections according to the cycle threshold (Ct) values. Reported Ct values >30 indicated either the absence of viral particles or the inhibition of the growth of infectious SARS-CoV-2 particles, with all viral RNA coming from the initial inoculation.

## Figures and Tables

**Table 1:** Mean Ct-values and standard deviations (SD) of RT-PCR performed from culture media after culture, and combined results from culture and PCR (pos = viral growth, neg = no viral growth). Different culture dilutions have been combined, and samples that were negative in RT-PCR have been excluded.

	1 min			5 min			10 min		
	Mean	SD	Result	Mean	SD	Result	Mean	SD	Result
**Sample 1**	16.08	0.58	pos	16.95	0.10	pos	16.40	1.29	pos
**Sample 2**	38.07	1.79	neg	NA	NA	neg	36.71	1.10	neg
**Sample 3**	16.60	0.45	pos	35.38	2.00	neg	36.03	2.76	neg
**Glass**	16.39	0.40	pos	21.06	9.36	pos	17.96	3.87	pos

NA indicates not applicable due to the absence of Ct value in the PCR in any replicates.

## Supplementary Materials

The following are available online at https://www.mdpi.com/article/10.3390/nano11071820/s1. Figure S1: Different plated surfaces with different colors as bronze (sample 1), dark brown (sample 2), and brown with surface imprinted COVIDSAFE making surface roughness (sample 3) delivered to the University of Helsinki to be tested for SARS-CoV-2 inhibition. Figure S2: Hydrophobic powder sample (sample 1) that painted the vial wall. Figure S3: TEM image (A) of the Cu-Ag nanohybrid powder sample (sample P), showing clumps of a broad range of different sizes, ranging from 26 nm Ag NPs to 1.3 ± 0.2 µm copper particles, and shapes (irregular rounded, rectangular, and flakes) of particles, with some particles acting like a cave enclosing other smaller particles inside them. Selected-area electron diffraction (SAED) ring pattern, depicting the crystalline Ag NPs mixed with Cu NPs. Figure S4: Ag map of Sample 2 demonstrates that the moon shaped particles are not Ag NPs (without the orange color used to identify the Ag). Video S1: Demonstration of the performance of the anti-SARS-CoV-2 surface (copper-silver nanohybrid plated surfaces) exposure tests in the BSL-3 laboratory. Table S1: EDX quantitative chemical composition of sample 2 in a concentration ascending order. Table S2: EDX quantitative chemical composition of sample 3 in a concentration ascending order. Table S3: Inhibitory effects of Cu-Ag nanohybrid powder sample (sample 1) and plated surfaces (samples 2 and 3) on SARS-CoV-2 after three time points based on the cytopathic effects. Two dilutions of each sample were cultured in two parallel reactions, and glass surfaces were used as a control. Table S4: Ct-values of RT-PCR performed from post-culture media of individual wells. Figure S5: Inhibitory effects of Cu-Ag nanohybrid plated surfaces (i.e., samples 1, 2, and 3) on the SARS-CoV-2 growth after time points of 1, 5, and 10 min based on RT-PCR performed from culture media. The error bars represent the standard deviation of the means from duplicates executed in BSL3. It is important to note the considerable error detected for the non-reactive glass samples that might be elicited from an unnoticed pipetting error.
