# Supplementary material for: Copper-Silver Nanohybrids: SARS-CoV-2 Inhibitory Surfaces
Source: Nanomaterials (Basel). 2021 Jul 13;11(7):1820. doi: 10.3390/nano11071820 (PMC8308209; doi:10.3390/nano11071820)
Supplement: Supplementary file 1 [file nanomaterials-11-01820-s001.zip › nanomaterials-1284550 Supplementary Material.pdf]

Copper-Silver Nanohybrids: SARS-CoV-2 Inhibitory Surfaces

Dina A. Mosselhy [1,2,] *, Lauri Kareinen [1,2], Ilkka Kivistö [1,2], Kirsi Aaltonen [1,2], Jenni Virtanen [1,2], Yanling Ge [3], Tarja Sironen [1,2,] *

[1] Department of Virology, Faculty of Medicine, University of Helsinki, P.O. Box 21, 00014 Helsinki, Finland; lauri.kareinen@helsinki.fi (L.K.); ilkka.kivisto@helsinki.fi (I.K.); kirsi.aaltonen@helsinki.fi (K.A.); jenni.me.virtanen@helsinki.fi (J.V.)

[2] Department of Veterinary Biosciences, Faculty of Veterinary Medicine, University of Helsinki, P.O. Box 66, 00014 Helsinki, Finland

[3] VTT Technical Research Center of Finland Ltd., P.O. Box 1000, FI-02044 VTT, Espoo, Finland; Yanling.Ge@vtt.fi

* Correspondence: dina.mosselhy@helsinki.fi (D.A.M.); tarja.sironen@helsinki.fi (T.S.)

Supporting Information

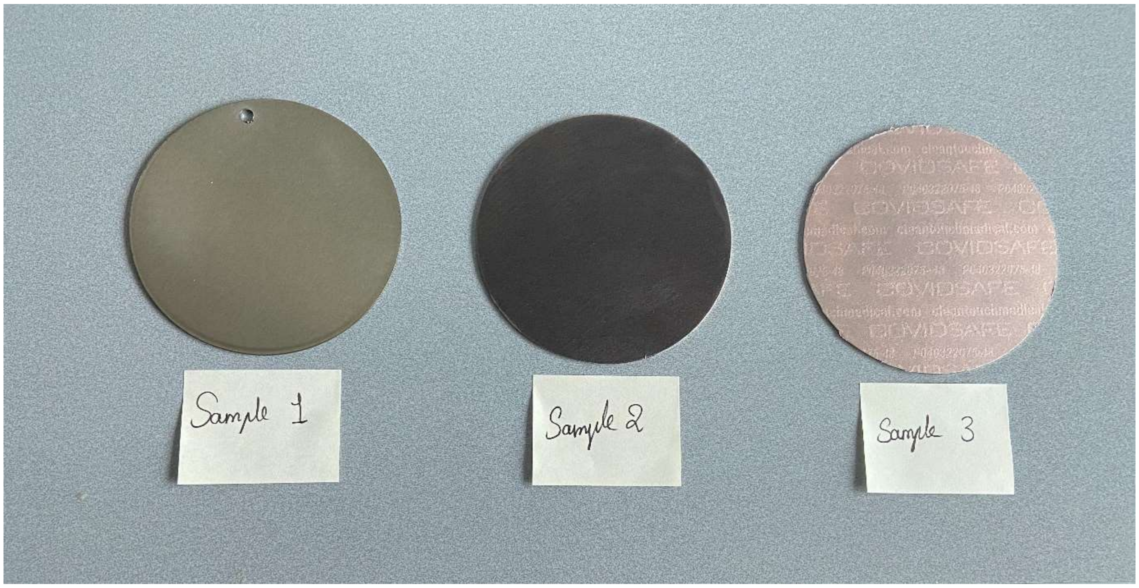

Figure S1. Different plated surfaces with different colors as bronze (sample 1), dark brown (sample 2), and brown with surface imprinted COVIDSAFE making surface roughness (sample 3) delivered to the University of Helsinki to be tested for SARS-CoV-2 inhibition.

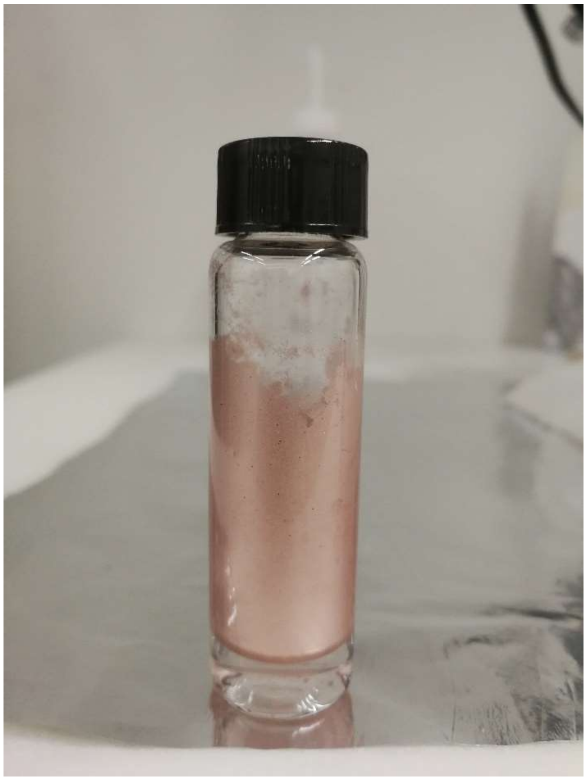

Figure S2. Hydrophobic powder sample (sample P) that painted the vial wall.

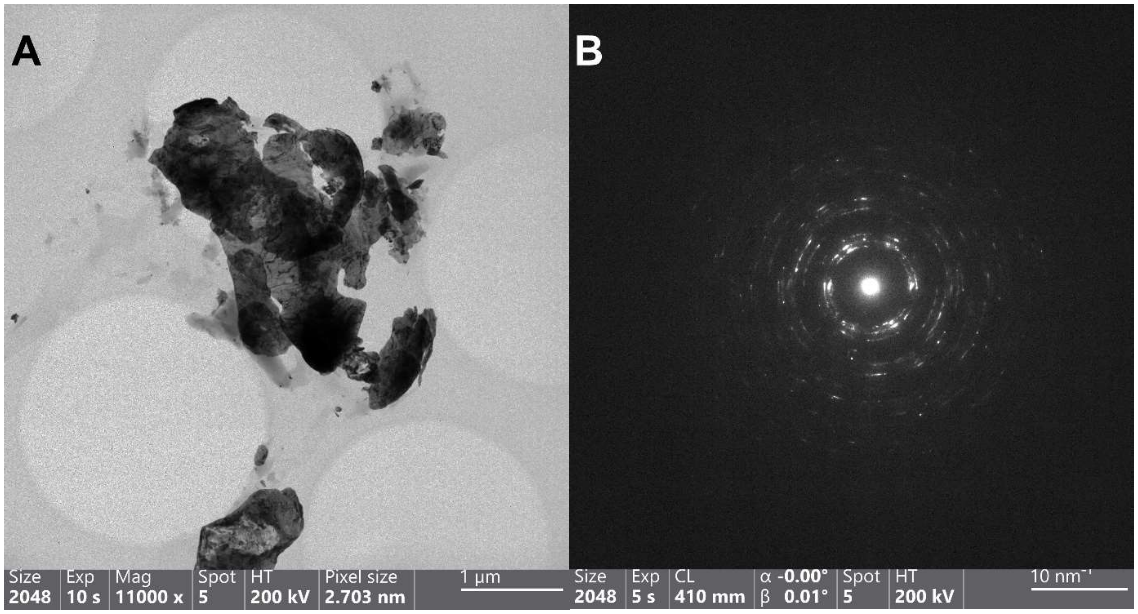

Figure S3. TEM image (**A**) of the Cu-Ag nanohybrid powder sample (sample P), showing clumps of a broad range of different sizes, ranging from 26 nm Ag NPs to 1.3 ± 0.2 µm copper particles, and shapes (irregular rounded, rectangular, and flakes) of particles, with some particles acting like a cave enclosing other smaller particles inside them. (**B**) Selected-area electron diffraction (SAED) ring pattern, depicting the crystalline Ag NPs mixed with Cu NPs.

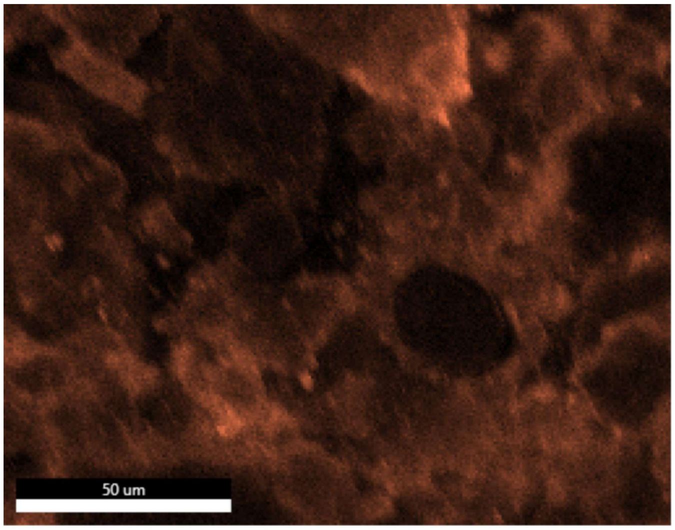

Figure S4. Ag map of Sample 2 demonstrate that the moon-shaped particles are not Ag NPS (without the orange color used to identify the Ag).

Table S1: EDX quantitative chemical composition of sample 2 in a concentration ascending order.

Element	Weight (wt)%	Atomic %	Error %
**SiK**	1.22	2.15	8.56
**AlK**	1.28	2.34	9.37
**TiK**	2.03	2.09	3.04
**SnL**	2.49	1.04	1.86
**AgL**	6.99	3.19	2.36
**ClK**	7.72	10.73	5.21
**NaK**	12.95	27.78	9.90
**CuK**	65.32	50.68	1.13

Table S2: EDX quantitative chemical composition of sample 3 in a concentration ascending order.

Element	Weight (wt)%	Atomic %	Error %
**FeK**	0.10	0.10	9.60
**TiK**	0.20	0.20	4.6
**SnL**	1.6	0.8	2.80
**SiK**	4.00	8.50	8.50
**AlK**	5.20	11.30	9.20
**AgL**	9.20	5.10	2.30
**CuK**	79.70	74.10	1.10

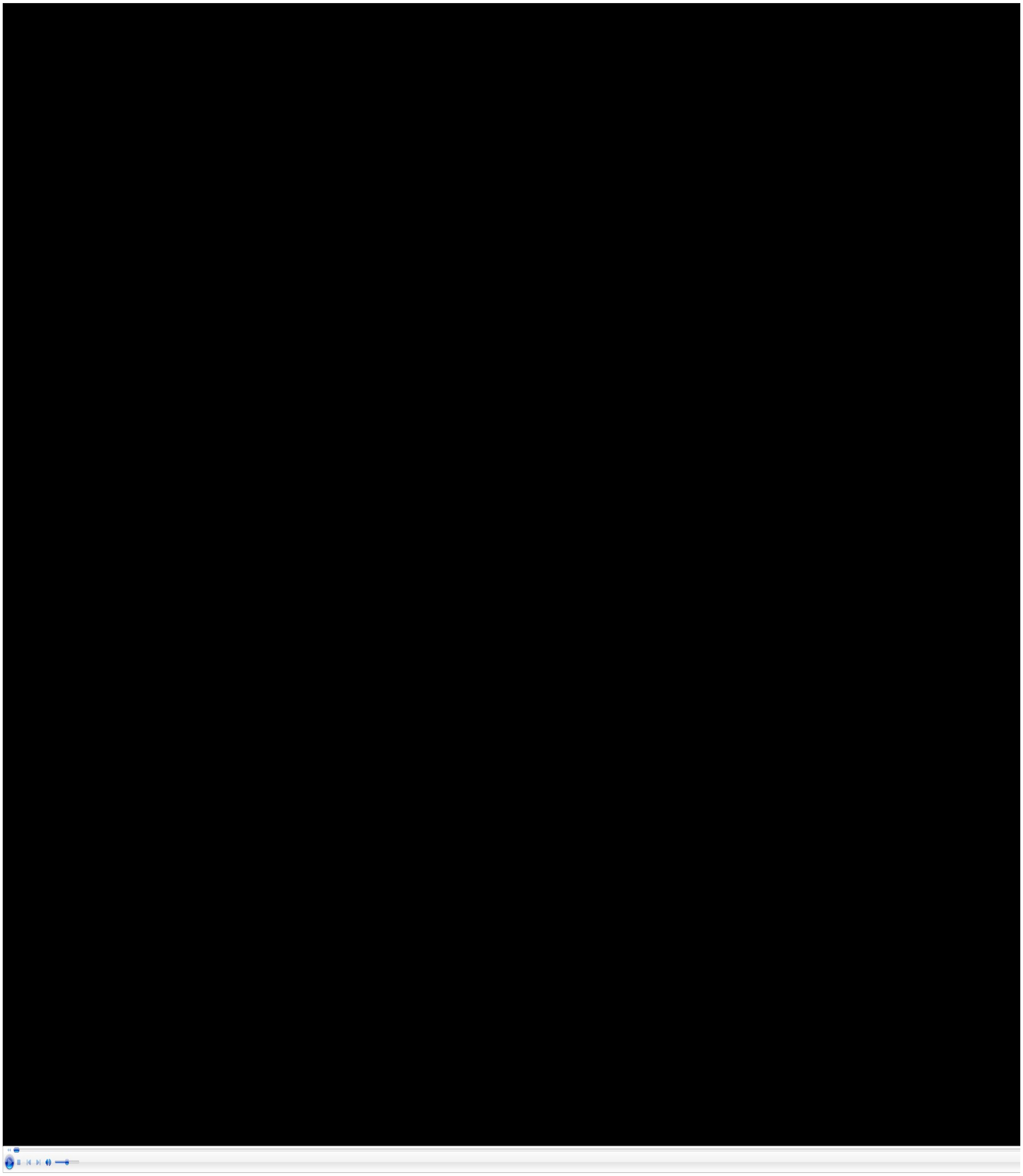

Video S1. Demonstration of the performance of the anti-SARS-CoV-2 surface (copper-silver nanohybrid plated surfaces) exposure tests in the BSL-3 laboratory.

Table S3. Inhibitory effects of Cu-Ag nanohybrid powder sample (sample 1) and plated surfaces (samples 2 and 3) on SARS-CoV-2 after three-time points based on the cytopathic effects. Two dilutions of each sample were cultured in two parallel reactions, and glass surfaces were used as a control. .

Sample	Dilution	1 min	5 min	10 min
**Sample 1**	1:1	+	+	+
	1:10	+	+	+
**Sample 2**	1:1	-	-	-
	1:10	-	-	-
**Sample 3**	1:1	+	-	-
	1:10	+	-	-
**Glass**	1:1	+	+	+
	1:10	+	+	+

+: Cytophatic effects observed on cells in both parallel wells

-: No cytopathic effects observed on cells in either of the parallel wells

Table S4. Ct-values of RT-PCR performed from post-culture media of individual wells.

Sample	Dilution	1 min		5 min		10 min	
	1:1	16.43	16.39	16.95	16.82	17,21	16,63
**Sample 1**	1:10	15.22	16.29	16.96	17.06	14,5	17,23
	1:1	38.9	35.85	No Ct	No ct	35,11	36,82
**Sample 2**	1:10	37.54	No ct	No Ct	No ct	37,4	37,49
	1:1	17.06	16.03	33.80	33.50	33,83	34,59
**Sample 3**	1:10	16.49	16.83	No Ct	37.11	35,68	No Ct
	1:1	16.97	16.07	16.49	15.43	16	16.06
**Glass**	1:10	16.29	16.21	35.05	17.26	16.02	23.76

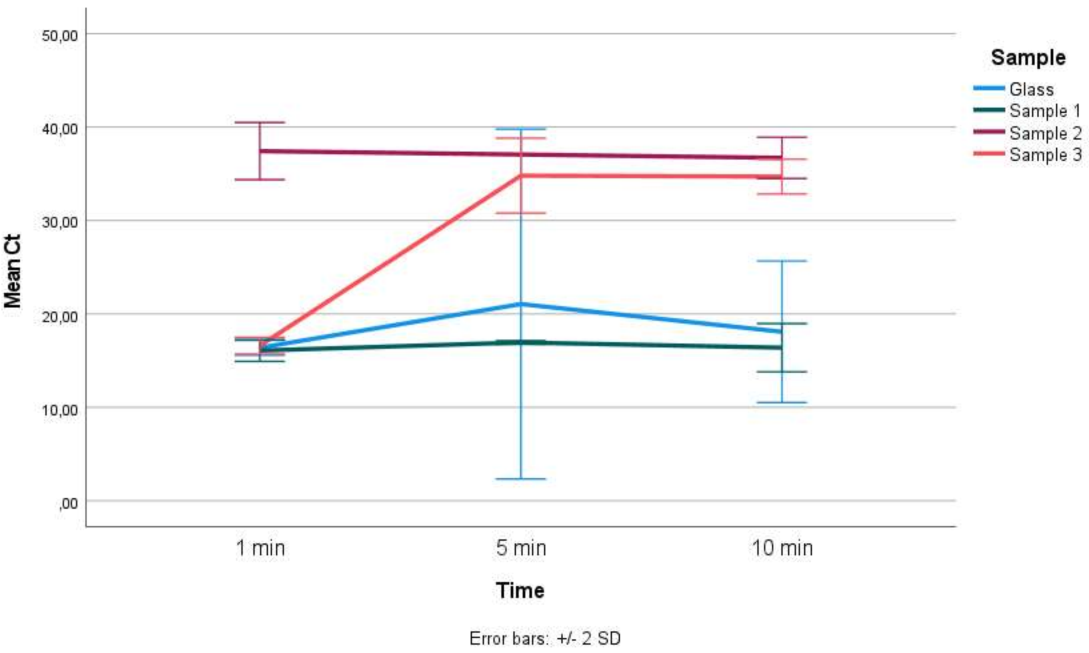

Figure S5. Inhibitory effects of Cu-Ag nanohybrid plated surfaces (i.e., samples 1, 2, and 3) on the SARS-CoV-2 growth after the time points of 1, 5 min, and 10 min based on RT-PCR performed from culture media. The error bars represent the standard deviation of the means from duplicates executed in BSL3. It remains good to notice the considerable error detected for the non-reactive glass samples that might be elicited from an unnoticed pipetting error.
